# Two Aspects of Activation: Arousal and Subjective Significance – Behavioral and Event-Related Potential Correlates Investigated by Means of a Modified Emotional Stroop Task

**DOI:** 10.3389/fnhum.2017.00608

**Published:** 2017-12-12

**Authors:** Kamil Imbir, Tomasz Spustek, Gabriela Bernatowicz, Joanna Duda, Jarosław Żygierewicz

**Affiliations:** ^1^Faculty of Psychology, University of Warsaw, Warsaw, Poland; ^2^Biomedical Physics Division, Institute of Experimental Physics, Faculty of Physics, University of Warsaw, Warsaw, Poland

**Keywords:** duality of activation mechanisms, emotional Stroop task, mechanisms of cognitive control, ERP, N450 P2

## Abstract

The arousal level of words presented in a Stroop task was found to affect their interference on the required naming of the words’ color. Based on a dual-processes approach, we propose that there are two aspects to activation: arousal and subjective significance. Arousal is crucial for automatic processing. Subjective significance is specific to controlled processing. Based on this conceptual model, we predicted that arousal would enhance interference in a Stroop task, as attention would be allocated to the meaning of the inhibited word. High subjective significance should have the opposite effect, i.e., it should enhance the controlled and explicit part of Stroop task processing, which is color naming. We found that response latencies were modulated by the interaction between the arousal and subjective significance levels of words. The longest reaction times were observed for highly arousing words of medium subjective significance level. Arousal shaped event related potentials in the 150–290 ms time range, while effects of subjective significance were found for 50–150, 150–290, and 290–530 ms time ranges.

## Introduction

### Emotional Stroop Tasks

Cognitive control is one of the most important mental abilities. It makes it possible for us to behave in a way that allows us to achieve our goals (Nigg, 2000; Cooper, 2010; Juvina, 2011). One of the best-known aspects of cognitive control is interference control, which is measured by the Stroop task (Stroop, 1935). Interference occurs when a task involves two conflicting processes (Nigg, 2000). The emotional Stroop test (EST) requires the subject to name the color in which the stimulus-word is printed. The words differ in the affective properties often described in terms of valence and arousal (Russell, 2003). In this test, the interfering processes are: (I) reading and accessing the meaning of the word, and (II) accessing the color in which the word is printed and naming the color. Process (I) is automated and habitual for fluent readers and process (II) is related to the explicit task. The efficiency of execution of the explicit task depends on the inhibition of the automated process. Response times on experimental trials (those with emotionally charged stimulus words) are compared to response times for control trials (those with emotionally neutral words).

Emotional Stroop test was found to be especially useful for determining the source of anxiety in individuals with clinical and subclinical symptoms of anxiety disorders (see Williams et al., 1996 for a review). Anxious individuals respond more slowly on trials where the meaning of the stimulus word is related to their clinical condition. This is because the meaning of relevant emotional words captures the respondent’s attention and it is harder to inhibit the predominant, automated reading response for relevant words than for irrelevant control words. Words connected with traumatic experiences are processed more deeply and thus response times on trials involving such words are longer (Watts et al., 1986; McKenna and Sharma, 1995, 2004).

Consistent with these results are findings suggesting that the EST effect is more robust and is evoked by affective features of external stimuli in populations with no particular trauma experienced (Nigg, 2000; Larsen et al., 2006). For example, the valence of words was found to modulate behavioral results. Responses to negatively valenced words were reported to be slower (cf. Williams et al., 1996; McKenna and Sharma, 2004) than responses to neutral words. EST effects for positively valenced words are reported to be much weaker (Pratto and John, 1991; Richards et al., 1992; McKenna and Sharma, 1995) and are more likely to be observed when the stimuli are of personal relevance to the respondent (Riemann and McNally, 1995). Such results were replicated recently for the emotional experience concept, describing subjective knowledge (low vs. high) of abstract words for emotions (Siakaluk et al., 2014). The main weakness of early EST investigations into valence effects is that the stimuli almost always permitted an interpretation based on factors other than those deliberately manipulated as part of the experimental design (Larsen et al., 2006).

### Factors Which Modulate EST Behavioral Effects

The investigation of factors responsible for slowdown in reaction latencies in EST showed that there are two important aspects of stimuli that modulate cognitive control effectiveness: word frequency in the language and arousal (Burt, 2002; Larsen et al., 2006). Words, which occur less frequently in natural language, yielded longer response times than more frequently occurring words. It is possible that this effect occurs because more cognitive resources are required to process less frequent words or because they are more surprising and therefore more likely to capture attention. Although frequency is not an affective dimension, these results corroborate the claim that the EST is a valid measure of inhibitory control (Nigg, 2000) related to various causes (relationship to personal trauma, personal relevance, knowledge, valence, or frequency).

Apart from frequency of usage, the arousal level of words was found to influence response latencies in EST independently of valence (Dresler et al., 2009). Highly arousing words are associated with longer response times on the color-naming tasks than less arousing words. Valence and arousal are related to one another by a U-shaped quadratic correlation, indicating that valenced words are more arousing than neutral words (e.g., Warriner et al., 2013; Imbir, 2015). It appeared that earlier results of valence could be accounted for by the differences in arousal properties of stimuli used in experiments. (Burt, 2002). Therefore, stimulus material has to be carefully selected in order not to compare more arousing negative words with less arousing positive words (Imbir and Jarymowicz, 2013).

### Event-Related Potential (ERP) Correlates of Word Processing and Stroop Tasks

When considering performance in the EST paradigm, two distinct types of event-related potential (ERP) correlates have already been identified. The first group contains manifestations of emotional word processing. They are referred to as the P1, N1, or P2. The second group reflects both the processes of involuntary word processing and interference control during the creation of responses to the task – they are referred as the P3 and N450 (Van Hooff et al., 2008). The P1 component typically has a latency of 80–130 ms and is maximal in the occipital areas (Hillyard et al., 1998; Van Hooff et al., 2008). The early timing and location suggest that P1 is related to early visual processing and P1 amplitude has been shown to be larger for attended than unattended stimuli (Hillyard et al., 1998). The valence of stimuli can also influence P1 amplitude, with negative words eliciting larger P1 responses than neutral words (Van Hooff et al., 2008). The N1 component is the next deflection and has been shown to be sensitive to the valence of words used in the emotional Stroop task (Pérez-Edgar and Fox, 2003), with anterior N1 responses to negative words being smaller than N1 responses to positive or neutral words.

The P2 component reaches its maximum amplitude at about 200–250 ms (Van Hooff et al., 2008) and in several studies, it has been shown to be sensitive to the emotional meaning of words. Unfortunately, the pattern of results associated with this component is rather inconsistent. Increases in amplitude relative to the P2 response to neutral words have been observed for positive words only (Schapkin et al., 2000), negative words only (Huang and Luo, 2006), or both positive and negative words (Carretié et al., 2004; Herbert et al., 2006). In the EST paradigm, threat-related words have been shown to elicit larger amplitude P2 responses than neutral words (Thomas et al., 2007).

The earliest component associated specifically with the EST is P3 (Metzger et al., 1997). In the EST, the P3 component has a centro-posterior localization and a latency of about 340–600 ms. P3 amplitude is greater for threatening words than neutral words, even without differentiation of reaction latencies between categories in healthy (no trauma reported) individuals (Thomas et al., 2007). The second component, which is sensitive to interference control in the Stroop task, is the N450 (West and Alain, 2000), which occurs about 350–500 ms after stimulus onset. This component is most pronounced in fronto-central locations, but may also take the form of a broadly distributed negativity (Van Hooff et al., 2008). N450 amplitude is greater on incongruent than congruent trials (West, 2003; West et al., 2004). It has been suggested that the N450 component reflects activation of the anterior cingulate cortex (Liotti et al., 2000), possibly associated with conflict detection (West, 2003, West et al., 2004) or the selection of competing responses (West and Alain, 1999). In the EST, the N450 has been shown to be sensitive to valence of stimulus words, with negative words eliciting larger N450 responses and slower behavioral responses (Van Hooff et al., 2008).

The interesting fact is that both reported EST components (P3 and N450) appear at the same time range, but show opposite polarities and distinct spatial localizations. The predominance of one or the other component may be related to the specificity of the experimental procedure. The study which reported that P3 was sensitive to the meaning of words on the EST (Thomas et al., 2007) included both word-reading and color-naming trials presented in random order and was thus a dual task. This may explain why P3 effect dominated over the measured activation (Luck, 2005; Thomas et al., 2007). Corroboration for this explanation comes from the finding that P3 was larger in word-reading trials than color-naming trials. In the study, which found that the N450 component was sensitive to stimulus valence (Van Hooff et al., 2008), the EST consisted solely of color-naming trials, which were presented in blocks of negative (incongruent condition) and positive (congruent) words.

### Activating Properties of Words: Duality of Activation Model

It could be anticipated that arousal, as an activating factor, should lead to facilitation of the processing, therefore to the shortening of reaction times. But, as has been discovered (cf. Burt, 2002), arousal plays a crucial role in the generation of behavioral slowdown observed in reaction times to EST trials. Duality of mind theories (see Gawronski and Creighton, 2013 for a review) state that there are two mechanisms underlying the processing of all stimuli, namely *automated* processes and *controlled* ones, each being specific to distinct mind systems: namely an experiential system and a rational one (Epstein, 2003). Arousal is a form of activation directly related to simple, highly automated processes of the experiential mind (Epstein, 2003; Imbir, 2016a). It enhances the processing that is crucial for survival (e.g., fight or flight reaction). An arousing stimulus captures the attention and induces activation to enable the organism to take action. Arousal has been shown to impair higher order processes like cognitive control (Nigg, 2000), because the processing and responding to arousing stimuli consumes all resources available for maintaining cognitive control (Kahneman, 2011).

The controlled processes of the rational mind have been proposed to have specific activation mechanisms (Imbir, 2015, 2016a). We can assume that the reflective (Strack and Deutsch, 2004, 2014) or rational (Epstein, 2003) mind uses propositional mechanisms, based on verbalization and rules of logic (Strack and Deutsch, 2014). This effortful form of processing is one that individuals tend to avoid (Kahneman, 2003, 2011). The important question is, why do people engage in such effortful propositional based thinking? This question concerns the nature of rational mind activation (Imbir, 2016b). This type of activation may rely on some form of resonance of the stimuli or situation with the goals, aims or value system of a person. This resonance causes people simply to perceive the situation as worthy of the effort, because it is critical to their goals and aims. In other words, it has subjective significance for them (Imbir, 2015, 2016a,c). Subjective significance is postulated to be activation for the rational mind. It can be operationalized as the perception of conscious willingness to exert effort in relation to situations or objects. Recently, a reliable measure of subjective significance (Imbir, 2015, 2016c) based on a modification of the Self-Assessment Manikin scale (Lang, 1980) was introduced. This measure consisted of the Self-Assessment Manikin scale (cf. Imbir, 2015) and its description stating that subjective significance varies from “experiences that are not significant to one’s goals, plans, and expectations, therefore they could be labeled by words such as trivial, gone unnoticed, fleeting, inconsequential, insignificant, unimportant” to experiences that are “very important to one’s goals, plans, and expectations, therefore they could be labeled by words such as vitally important, significant, turning-point, consequential, meaningful, decisive” (Imbir, 2015, **Table 1**, p. 865). It appeared, that assessments of both arousal and subjective significance made by participants for a large number of Polish words (Imbir, 2016c) revealed the independence of those two dimensions (in terms of low correlations between them) as well as comparable and high reliability of both dimensions (in terms of repeatability of assessments; for review see Imbir, 2015, 2016c). Just as there are stimuli which elicit arousal, there are also stimuli which elicit subjective significance associations for a given individual. It appeared also that subjective significance is similar to arousal in terms of the possibility of treating subjective significance as some form of universal association for certain stimuli in a certain age group living in specific conditions. For example, the word *graduation* bears a certain connotation of subjective significance, especially when students are approaching this moment. In other words, just as in the case of arousal, some stimuli possess the potency to evoke rational mind activation due to their appearance, and thus, may be used in the EST paradigm (Imbir, 2016b). **Table 1** presents examples of words for the four extremes of low/high arousal × low/high subjective significance dimensions.

**Table 1:** Examples of low/high arousing × low/high subjective significant words used as experimental manipulation.

		Arousal level	
		Low	High
Subjective	Low	*prasowanie (ironing)*	*galop (gallop)*
significance		*seria (series)*	*hazard (gamble)*
level		*orbitowanie (orbit)*	*strzał (shot)*
		*aspekt (aspect)*	*zombie (zombie)*
		*zero (zero)*	*koszary (barracks)*
		*sfinks (sphinx)*	*wolt (volt)*
		*echo (echo)*	*wampir (vampire)*
		*tenor (tenor)*	*szaleństwo (craze)*
		*namiot (tent)*	*smok (dragon)*
		*pauza (pause)*	*harem (harem)*
		*wersja (version)*	*karykatura (pamphlet / caricature)*
		*klan (clan)*	*parada (parade)*
		*pasmo (band)*	*kryminał (thriller / jail)*
		*smuga (streak)*	*salwa (salvo)*
		*mila (mile)*	*car (tsar)*
	High	*poezja (poetry)*	*geniusz (genius)*
		*wykonywanie (implementing)*	*poprawka (amendment)*
		*krok (step)*	*majątek (fortune)*
		*zdanie (sentence/sense)*	*zwycięzca (winner)*
		*osoba (person)*	*maniak (maniac)*
		*istota (being)*	*płomień (flame)*
		*godzina (hour)*	*alarm (alert / alarm)*
		*singiel (single)*	*sesja (session)*
		*czyn (deed)*	*ogień (fire)*
		*jednostka (unit)*	*buntownik (rebel)*
		*emerytura (pension)*	*ekstrawertyk (extrovert)*
		*lekcja (lesson)*	*burza (storm)*
		*zasada (rule)*	*wysiłek (effort)*
		*dokument (document)*	*wynik (result)*
		*woń (odor)*	*władza (authority)*

### The Interplay between Arousal and Subjective Significance Factors in EST: Behavioral Evidence

The impact of the factors discussed above, related to the concept of activation, were measured in a series of two behavioral studies involving a modified Stroop task (MST; Experiment 1) and classical Stroop test merged with random presentation of words (printed in black) differing in arousal and subjective significance (Experiment 2; cf. Imbir, 2016b). The MST paradigm was constructed in the same way as the EST. The only difference was that in case of MST the activation factors were manipulated while all stimuli were controlled for neutral valence (cf. Materials and Methods section). The task was to name the color of the letters for words meaning colors, but no specific instruction was given for activation related words. It appeared that reaction latencies were longer for highly arousing words compared to low arousing words, but only when subjective significance was moderate. The presence of subjective significance (both low and high) effected a reduction of slowdown caused by highly arousing words (Imbir, 2016b).

**FIGURE 1:**
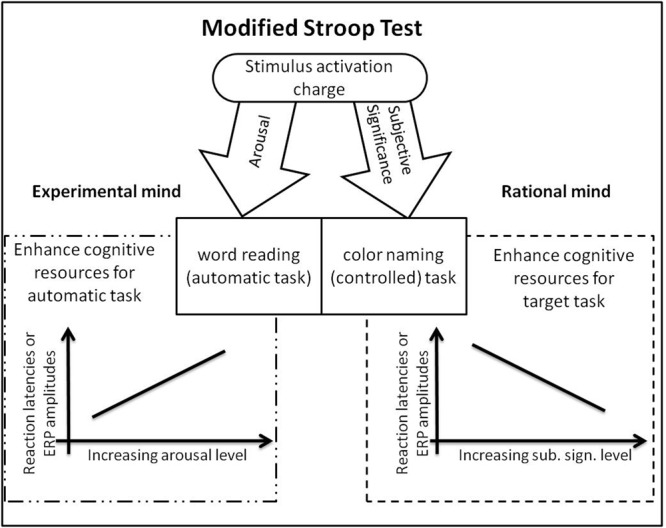
Activational properties of stimuli (arousal and significance) and their influence on Stroop task components and behavior (Imbir, 2016b).

To understand this pattern of results, we have to refer to the dual process nature of the Stroop task (Imbir, 2016b). We posit that behavioral responses in the Stroop task involve two distinct processes (see **Figure 1**) specific to the experimental and the rational mind, respectively. The first is the involuntary, automatic reading of the word stimulus. Increased allocation of resources for this process is directly responsible for the increase in response latencies observed when the arousal level of stimuli is high. The second process is related to the explicit task, i.e., naming the color in which the words are displayed. Successful execution of the explicit task requires suppression of the automatic process and allocation of the resources for color naming. This is effortful, because it is unusual to do this while reading. Cognitive control is needed to ignore the habitual, automatic action and to focus on executing the explicit task. This control should be augmented when the meaning of the stimulus word has subjective significance, because subjective significance should enhance the motivation to engage in effortful processing. In summary, arousing stimuli, which activate the experiential mind, should enhance automated processing whereas subjectively significant stimuli activate the rational mind and hence enhance controlled processing. This conceptual model is presented in **Figure 1**.

Recently, in the literature, there is a debate over the interpretation of the results for the Stroop test paradigm, and the EST in particular, in terms of attention shift rather than cognitive control (e.g., Algom et al., 2004; Ben-Haim et al., 2014). In particular, behavioral effects in EST paradigms may be triggered by the allocation of attention to affective manning of stimuli presented to participants. If so, we may assume that arousal may focus attention on the automatic part of a task (reading), while subjective significance may focus attention on the controlled aspect of a task (color naming). Expected results are the same as presented in **Figure 1**. The current experiment was not designed in order to validate the control or attention explanations for the EST phenomenon, but to highlight the fact that activation associated with words may not just disrupt the effectiveness of responses. It may also enhance it (cf. Imbir, 2016b).

### Aim and Hypothesis

The main aim of this study was to investigate how the arousal and subjective significance of stimuli influence performance on a MST on the level of electrophysiological correlates of their influence. Behavioral results (Imbir, 2016b) revealed partial following of the predictions depicted in **Figure 1**, namely low subjective significant stimuli were found to reduce the slowdown caused by high arousal level in the same way as high subjective significant stimuli. The interpretation of this effect on the basis of behavioral data appeared to be difficult, thus we decided to investigate ERP correlates allowing verification of the timing of effects caused by both activation factors. Due to the exploratory stage of investigation, we decided to analyze all the components of ERP measured during MST performance.

The simple predictions from the model depicted in **Figure 1** are that a pure increase in the arousal level of stimuli should make reaction latencies longer and simultaneously should increase the absolute values of ERP amplitudes in MST, while a pure increase in the subjective significance of levels of stimuli should make the reaction latencies shorter and the absolute values of ERP amplitudes should decrease. Pure effects are expected to appear in behavioral data and distinct time ranges of ERP when only one factor is taken into consideration.

## Materials and Methods

### Participants

Thirty-two individuals (women = 16, men = 16) aged from 19 to 26 years (*M* = 21.63, *SD* = 1.98) participated in the study. The participants were students at various Warsaw colleges and universities and participated voluntarily in return for a small reward. All the participants were right handed, native Polish speakers with normal or corrected-to-normal vision. Participants provided verbal informed consent to participation in the presence of at least two members of the research team and documented in a research diary; we did not get written consent as we had assured the participants of anonymity. The bioethical committee responsible for approving the research suggested this procedure. We did not collect any personal data on our participants. The bioethical committee of the Maria Grzegorzewska University approved the design, experimental conditions, and consent procedure for this study. All procedures performed in studies involving human participants were in accordance with the ethical standards of the institutional and/or national research committee and with the 1964 Helsinki declaration and its later amendments or comparable ethical standards.

### Design

In this study, we investigated the behavioral and electrophysiological correlates of the reading of emotional words. We manipulated the arousal levels of the words (three levels) and their subjective significance (three levels) whilst ensuring that stimuli were matched with respect to valence, concreteness, frequency of appearance in language and length. Data on response accuracy and number of correct and artifact-free trials were not normally distributed so effects relating to these variables were assessed using the Friedman test for replicated block designs. Other effects were assessed using repeated measures ANOVA. Mauchly’s test was used to assess the assumption of sphericity, and Greenhouse–Geisser correction for violations of sphericity was used where necessary. Statistical significance in multiple comparisons was corrected by Holm’s procedure (Holm, 1979).

### Linguistic Material

Because the affective properties of the stimulus words are crucial to the MST task (Larsen et al., 2006), they must be chosen carefully if the task is to yield a measurement of the interference effect. The experimental and control words should differ with the respect to the dimension of interest (e.g., arousal) whilst be matched with respect to all other potentially important factors (e.g., valence or frequency of usage). The stimulus words were 135 nouns with known affective properties selected from a set of 4905 words from Affective Norms for Polish Words Reloaded (Imbir, 2016c) which was compiled using the same methodology used previously to produce a smaller collection of affective norms (Imbir, 2015). We manipulated two activation dimensions of the stimuli: arousal and subjective significance and matched the stimulus sets with respect to affective dimensions such as valence, concreteness and lexical properties such as frequency of appearance in Polish (based on Kazojć, 2011) and word length (number of letters). The parameters for all stimulus words used in this study are presented in Appendix 1. **Table 2** presents descriptive statistics (*M* and *SD*) for arousal and subjective significance.

**Table 2:** Word properties for each group of words calculated on the basis of Affective Norms for Polish Words Reloaded (Imbir, 2015, 2016b).

			Arousal					
			Low		Medium		High	
			*M*	*SD*	*M*	*SD*	*M*	*SD*
Significance	Low	Arousal	3.20	0.29	3.84	0.27	4.79	0.53
		Significance	2.87	0.33	2.96	0.18	2.93	0.60
		Valence	5.25	0.48	5.11	0.44	5.01	0.63
		Concreteness	4.07	1.02	3.88	0.73	3.91	0.90
		LN frequency	6.04	1.86	6.32	1.41	5.95	1.36
		Length	5.73	2.09	6.20	1.32	6.07	2.02
	Medium	Arousal	3.20	0.16	3.85	0.26	4.85	0.35
		Significance	3.56	0.32	3.71	0.22	3.74	0.34
		Valence	5.42	0.47	5.38	0.62	5.10	0.66
		Concreteness	3.92	0.90	3.94	0.90	3.98	0.75
		LN frequency	6.50	1.52	6.29	1.58	5.68	1.91
		Length	6.27	2.09	6.13	1.68	6.87	2.45
	High	Arousal	3.27	0.27	3.85	0.32	4.97	0.33
		Significance	4.55	0.31	4.64	0.40	4.88	0.44
		Valence	5.38	0.36	5.41	0.35	5.31	1.11
		Concreteness	4.28	0.75	4.33	1.00	4.37	0.98
		LN frequency	6.99	2.02	7.08	1.23	6.55	1.90
		Length	6.47	2.13	6.67	1.95	6.87	2.00

*Participants in the normative sample made their ratings on each dimension by using a 9 point Likert scale implemented as varying expressions of a self-assessment manikin (SAM). LN frequency = logarithm natural transformation of frequency.*

To check that our stimulus sets differed as planned with respect to their arousal and subjective significance we used ANOVA. The dependent variables were arousal ratings or subjective significance ratings, factors were arousal (three levels) and subjective significance (three levels). Words (135 items) played the role of observations. For arousal ratings, there was a main effect of arousal level, *F*(2,126) = 31.09, *p* < 0.001, η^2^= 0.83, but no main effect of subjective significance level, *F*(2,126) = 0.88, *p* = 0.4, η^2^= 0.01 nor an interaction *F*(4,126) = 0.3, *p* = 0.9, η^2^= 0.009. For subjective significance ratings, there was no main effect of arousal level, *F*(2,126) = 3.02, *p* = 0.053, η^2^= 0.04; but there was a main effect of subjective significance level, *F*(2,126) = 35.62, *p* < 0.001, η^2^= 0.81. Although the *p-*value for the effect of arousal level was only slightly above 0.05 there was a huge difference between the value of η^2^ for arousal level and subjective significance level, so we concluded that the manipulation was effective and that the two factors were independent. There was no interaction between arousal level and subjective significance level: *F*(4,126) = 0.73, *p* = 0.6, η^2^= 0.023.

To ensure that words chosen differed only with respect to the parameters we intended to manipulate we also performed additional 3 (arousal level) × 3 (subjective significance level) ANOVAs for ratings of valence, ratings of concreteness, natural logarithm of frequency, and word length as dependent variables. There was no main effect of arousal level, subjective significance level or interaction of both on valence ratings, *F*(2,126) = 1.46, *p* < 0.23, η^2^= 0.02; *F*(2,126) = 1.89, *p* = 0.16, η^2^= 0.03 and *F*(4,126) = 0.23, *p* = 0.9, η^2^= 0.007, respectively. There was also no main effect of arousal level, subjective significance level or interaction of both on concreteness, *F*(2,126) = 0.03, *p* = 0.97, η^2^< 0.001; *F*(2,126) = 2.74, *p* = 0.07, η^2^= 0.04 and *F*(4,126) = 0.12, *p* = 0.9, η^2^= 0.004, respectively. Because the frequency data showed positive skew we analyzed natural logarithm-transformed data from the Kazojć (2011) database. There was no main effect of arousal level, subjective significance level or interaction of both on word frequency, *F*(2,126) = 1.24, *p* = 0.29, η^2^= 0.02; *F*(2,126) = 2.99, *p* = 0.054, η^2^= 0.05 and *F*(4,126) = 0.19, *p* = 0.95, η^2^= 0.006, respectively. Finally, there was also no main effect of arousal level, subjective significance level or interaction of both on word length, *F*(2,126) = 0.57, *p* = 0.57, η^2^= 0.01; *F*(2,126) = 1.29, *p* = 0.28, η^2^= 0.02, and *F*(4,126) = 0.19, *p* = 0.94, η^2^= 0.006, respectively.

These analyses demonstrated that our manipulation was effective in creating sets of words, which could be dissociated according to their arousal and subjective significance. We also successfully matched the sets of words with respect to other factors that might influence performance on the Stroop task, namely frequency of appearance, word length, valence and concreteness; thus, any differences observed could be firmly attributed to the manipulated variables.

### Procedure

Participants sat in a comfortable chair facing a 15-inch computer screen at a distance of approximately 1 m. Stimulus words were presented in 50-point Helvetica font using experimental software implemented in Python. Words were presented in four different colors: orange [decimal code (R,G,B): 255,143,65), red (200,0,0), green (0,200,0), and blue (0,0,255)]. When a stimulus word was being displayed four letters (P, C, Z, N, respectively *Pomarańczowy, Czerwony, Zielony*, and *Niebieski*) representing possible answers (initial letters of color names in Polish) were displayed at the bottom of the screen in the same order across trials. Participants were required to choose and press one of the four keys “c”, “v”, “b”, and “n” that were marked by P, C, Z, N letters printed in black on white stickers and placed over keys. Participants were encouraged to respond with their right hand keeping the fingers on the marked keys (located near to the Space key). This minimized the need for eye movement to visually control the response. Before the main part of the experiment, the participant completed a training session using the standard Stroop test (Stroop, 1935). This session consisted of 20 practice trials in which the participant was required to name the color of bars or squares displayed in one of four colors or read color words, followed by 60 standard trials in which the participant had to name the color in which color words (congruent or incongruent; random order) were displayed. Participants were encouraged to respond as quickly and accurately as possible. This training session was used to ensure that participants understood the task and were able to perform it correctly.

**FIGURE 2:**
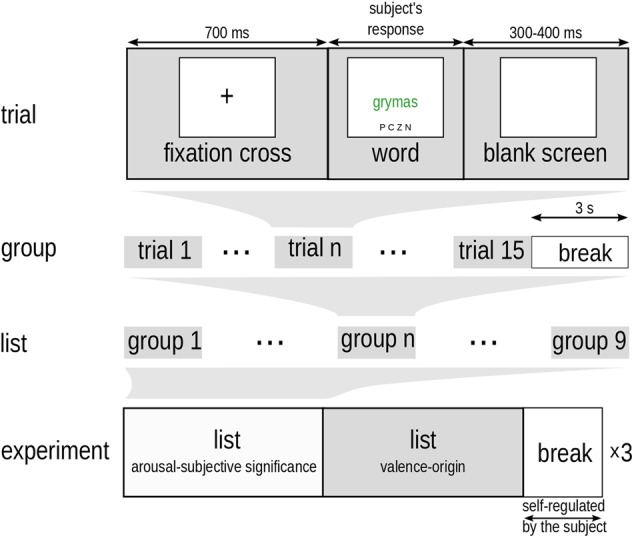
Outline of the experimental protocol.

In experimental trials, the task was to indicate the font color in which activation charged words were displayed. A schematic diagram of the experimental protocol is provided in **Figure 2**. At the start of a trial, a fixation cross was presented for 700 ms. This was followed by presentation of the stimulus word, which remained on the screen until the participant responded. This triggered the start of a 300–400 ms inter-trial interval during which the screen was blank. Stimuli were presented in homogenous 15-word blocks. There were nine types of trial corresponding to all the possible combinations of manipulation factors. We used a block design based on evidence that EST effects are especially pronounced with this type of procedure (cf. Bar-Haim et al., 2007). The subject could rest for 3 s after the presentation of each group. There were altogether nine groups, one for each possible combination of factor levels (3 arousal levels × 3 subjective significance levels), comprising a list of nine groups (9 × 15 = 135 words). The order of groups on the list, as well as the order of words within each group, was randomized. A measurement session had three repetitions of 135 words lists separated by a longer break with duration that was self-regulated by the subject. This means that single group of words (e.g., high arousing of high level of subjective significance associations) consisted of 45 trials (3 × 15). When considering main effects, a single group of words (e.g., low arousing) consisted of 135 trials (3 × 45).

The whole experimental procedure was composed of three repetitions of two distinct lists of words. Each repetition consisted of presentation of the 135-word experimental list described above, alternating with presentation of a different 135-word list, which constituted the material for a second experiment concerning the role of valence and origin of an affective state associated with words for EST performance (Imbir et al., 2017). The 135 items from the second list contrasted orthogonally three levels of valence (negative, neutral, positive) and three levels of origin of an affective state (automatic, control, reflective), factor claimed to be emotional manifestation of dual minds (cf. Imbir, 2016a). Also, arousal, concreteness, frequency of appearance, and length were controlled. The order of lists presentations was randomized between subjects.

### EEG Recording and Analysis

#### Apparatus

Stimuli were displayed on a standard personal computer monitor (LCD display; 15-inch diagonal). A second personal computer was used for monitoring and recording EEG data. Stimuli and EEG data were synchronized using a custom-made hardware trigger^1^. EEG activity was recorded from 19 electrode sites, Fz, Cz, Pz, Fp1/2, F7/8, F3/4, T7/8, C3/4, P7/8, P3/4, O1/2, referenced to linked earlobes, grounded on the clavicle and with impedances of 5 kΩ or less. Additionally, vertical EOG was recorded from an electrode above the left eye referenced to linked ears. We used Ag/AgCl electrodes. The signal was acquired using a Porti7 (TMSI) amplifier with a sampling frequency of 256 Hz. SVAROG^2^ was used for the EEG data monitoring and recording.

#### Offline EEG Signal Processing

The offline processing of the signal was performed in Matlab^®^ with the EEGLAB (Delorme and Makeig, 2004) toolbox. The signal was zero-phase filtered with Butterworth high- and low-pass filters (2nd order, corresponding to 12 dB/octave roll-off, with half amplitude cut-off frequency = 0.1 Hz and 30 Hz, respectively), and with an IIR notch filter at 50 Hz, to remove line noise. Epochs from 200 ms before stimulus onset to 850 ms post-stimulus onset were extracted and baseline-corrected (baseline data taken from -200 ms to 0 ms).

After exclusion of error trials and trials contaminated by artifacts (e.g., eye blinks or muscle activity, assessed by visual inspection of EEG and EOG recordings) the mean number of trials across condition was 37 (*SEM* = 0.3, *MIN* = 36.5, *MAX* = 37.9). The Friedman test for replicated block designs indicated that mean number of trials per condition was similar for the arousal groups with subjective significance as a blocking variable [χ^2^ = 4.4, *p* = 0.1] and for the subjective significance groups with arousal as a blocking variable [χ^2^ = 0.47, *p* = 0.8]. ERPs were averaged across trials, according to the experimental conditions.

## Results

### Behavioral Data

Response latencies were measured as the interval between stimulus onset and the pressing of a response key by the participant. Incorrect trials and those with extreme response latencies, i.e., those in the bottom or top 2.5 percent of the distribution, were excluded from the analysis (in practice included latencies ranged from 387 to 2121 ms). Natural logarithm-transformed response latencies were analyzed using a 3 (arousal levels) × 3 (subjective significance levels) repeated measures ANOVA. It is standard procedure to subject reaction time data to a natural logarithm transformation in order to transform the right-skewed distribution into an approximation of the normal distribution (cf., Heathcote et al., 1991) and thus enable parametric statistics to be used. **Table 3** presents response latencies in ms for each combination of arousal and subjective significance. There were main effects of arousal level: *F*(2,62) = 3.60, *p* < 0.03, subjective significance level, *F*(2,62) = 3.13, *p* < 0.05, and an interaction between arousal level and subjective significance levels, *F*(4,124) = 2.62, *p* < 0.04. The response latency for highly arousing stimuli was longer than for moderately arousing stimuli [*t*(31) = 2.79, *p* < 0.03]. The subjective significance effect reflected the fact that the response latency was longer for moderately significant stimuli than for highly significant stimuli [*t*(31) = 2.11, *p* < 0.06], and minimally significant stimuli, [*t*(31) = 2.20, *p* < 0.06]. Further *post hoc* pairwise *t*-tests using the Holm correction for multiple comparisons (adjusted *p*-value) indicated that the effect was due to the fact that response latencies for moderately significant, highly arousing stimuli (*M* = 869 ms; cf. **Table 3**) were longer than for other combinations of arousal and subjective significance, with the exception of moderately significant and low arousing (*M* = 834 ms) and low significant highly arousing (*M* = 821 ms).

**Table 3:** Mean and median response times (in ms) for each condition: the shading illustrates the interaction between conditions.

		Arousal			
		Low *M* (*SEM*) *MED*	Medium *M* (*SEM*) *MED*	High *M* (*SEM*) *MED*	Total *M* (*SEM*) *MED*
Subjective Significance	Low	806 (27)	815 (26)	822 (29)	814 (16)
		766	794	781	785
	Medium	834 (31)	803 (25)	869 (32)	835 (17)
		791	770	858	809
	High	812 (27)	810 (28)	812 (29)	811 (16)
		766	765	765	765
	Total	817 (16)	809 (15)	834 (17)	820 (9.4)
		773	772	808	780

*Response times in the condition marked in dark gray were significantly longer than the response times in conditions marked in light gray.*

The mean percentage of correct responses was 91% (*SEM* = 0.3). The Friedman test for replicated block designs indicated that accuracy was similar for all levels of arousal with subjective significance as a blocking variable [χ^2^ = 0.139, *p* = 0.933] and for all levels of subjective significance groups with arousal as a blocking variable [χ^2^ = 0.012, *p* = 0.994].

### Electrophysiological Data

#### Selection of Time Windows and Regions of Interest (ROIs)

**FIGURE 3:**
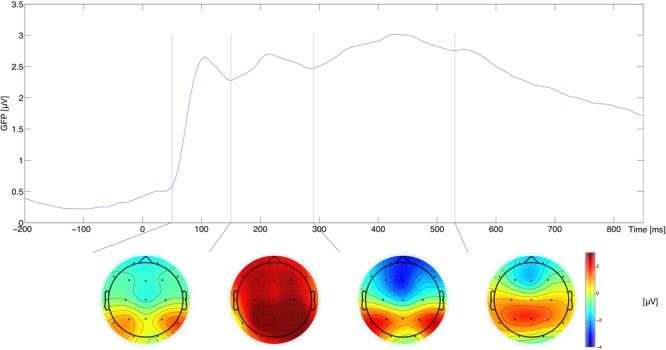
Global field power (upper plot) and topographies of average amplitude for a given time-window (bottom plot). The vertical lines in the upper plot indicate time window boundaries.

The components were defined as occurring in the following time windows: 50–150 ms, 150–290 ms, 290–530 ms, and 530–850 ms, based on the timing of successive maxima in the global field power (GFP) curve (**Figure 3**; the successive microstates are depicted as topographical distributions of amplitude averaged across the corresponding time window, conditions, and subjects). GFP is computed as spatial standard deviation, and quantifies the sum of electrical activity over all electrodes at a given time point. The latencies of GFP maxima indicate the latencies of evoked potential components (Lehmann and Skrandies, 1980; Skrandies, 1990).

The ROIs were selected to give us the opportunity to investigate the antero-posterior distribution of effects. These are: frontal (F: electrodes F3, Fz, F4); central (C: electrodes C3, Cz, C4); parietal (P: electrodes P3, Pz, P4). Signals from electrodes within each ROI were averaged. A similar approach has been used in other research into the neural correlates of the EST task (e.g., Schirmer and Kotz, 2003; Thomas et al., 2007; Taake et al., 2009). For the sake of completeness of presentation Appendix 2 summarizes grand average ERPs for individual electrodes and simple contrasts between levels of factors.

#### ERP Amplitudes

The effects of manipulation of factors were evaluated statistically by applying a three-factor repeated measure analysis of variance (significance × arousal × ROI) to the mean amplitude from each subject, in each of the time windows. For each of the time windows there was a significant main effect of ROI but this finding is not interesting and will not be discussed further. We did not obtain significant interactions between ROI and arousal [*F*(4,124) = 1.72, 𝜀**_GG_** = 0.453, *p_corr_* > 0.19, in time window 290–530 ms; in other time windows the effect was less significant] nor between ROI and subjective significance, [*F*(4,124) = 2.39, 𝜀**_GG_** = 0.568, *p_corr_* > 0.09, in time window 290–530 ms; in other time windows the effect was less significant] therefore further we report results for the amplitudes averaged across all ROIs. We did not obtain any interaction effects between arousal and subjective significance (**Table 4**).

**Table 4:** Statistics for interaction effects of arousal and subjective significance on mean amplitudes in the analyzed time windows.

Time window (ms)	*F*(4,124) statistics	Greenhouse–Geisser epsilon	Corrected *p*
50–150	0.710	0.847	0.57
150–290	1.692	0.830	0.17
290–530	0.212	0.918	0.92
530–850	0.805	0.824	0.50

##### Effects related to arousal

**FIGURE 4:**
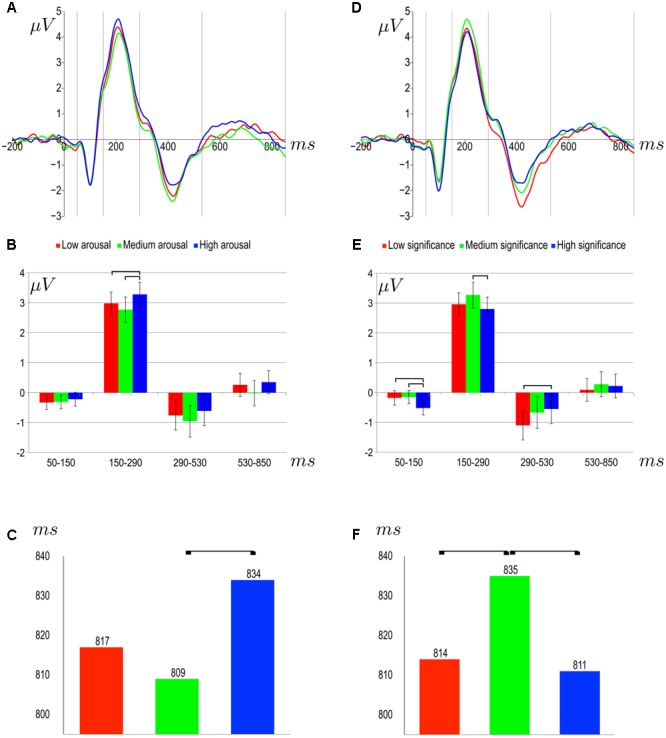
Event-related potential (ERP) results. Top: grand average (across subjects and regions of interest ROIs)] amplitude of ERPs: **(A)** for different levels of arousing properties; **(D)** for different levels of subjective significance. Level is indicated by color according to the legend. The vertical lines mark time window boundaries. Horizontal axis – time in ms; vertical axis - amplitude in μV. Middle: average amplitudes of components (with standard error indicated) in consecutive time windows for: **(B)** different levels of arousing properties; **(E)** different levels of subjective significance. Significant differences are indicated with square brackets. Amplitude in μV is represented on the vertical axis. Bottom: average response time in ms **(C)** different levels of arousing properties; **(F)** different levels of subjective significance.

The only main effect of arousal was obtained in the 150–290 ms time window, *F*(2,62) = 8.19, *p* = 0.0007. The amplitude of this potential was more positive for highly arousing stimuli (*M* = 3.28 *SEM* = 0.40) than for moderately arousing stimuli (*M* = 2.77, *SEM* = 0.42; *t*(31) = 3.64, *p* = 0.003), and for low arousing stimuli (*M =* 2.98, *SEM* = 0.38; *t*(31) = 2.87, *p* = 0.01). Grand average ERPs for the various levels of arousal are shown in **Figure 4A** and the differences between conditions are summarized in **Figure 4B**.

##### Effects related to subjective significance

There was a main effect of subjective significance on the amplitude of the potential in the time window 50–150 ms, *F*(2,62) = 6.69, *p* = 0.002. The amplitude in response to highly significant stimuli (*M* = -0.52, *SEM* = 0.23) was more negative than in response to minimally significant stimuli (*M* = -0.18, *SEM* = 0.24; *t*(31) = 3.05, *p* < 0.01) or moderately significant stimuli (*M* = -0.15, *SEM* = 0.22; *t*(95) = 2.85, *p* < 0.01).

In the next time window, 150–290 ms, there was also a main effect of subjective significance, *F*(2,62) = 4.64, *p* = 0.015. The amplitude of the potential related to moderately significant stimuli (*M* = 3.27, *SEM* = 0.43) was more positive than that related to highly significant stimuli (*M* = 2.80, *SEM* = 0.40; *t*(31) = 3.39, *p* = 0.006). In the 290-530 ms time window there was once again a main effect of subjective significance, *F*(2,62) = 4.43, *p* = 0.016. The amplitude of the potential was more negative for minimally significant stimuli (*M* = -1.10., *SEM* = 0.49) than for highly significant stimuli (*M* = -0.55, *SEM* = 0.49; *t*(31) = 3.05, *p* = 0.01). There was no main effect of subjective significance on the amplitude of the response in the 530–850 ms time window. Grand average ERPs for the various subjective significance levels are shown in **Figure 4D**. The differences between conditions are summarized in **Figure 4E**.

## Discussion

The main aim of this study was to investigate how two aspects of activation, namely arousal and subjective significance, influence interference cognitive control measured in a modified Stroop test. The MST used was very similar to the standard EST. The only difference was that rather than varying the valence of the stimulus words (they were all of neutral valence) we manipulated the activation properties of the stimuli by carefully selecting stimuli representing three levels of arousal and three levels of subjective significance (Imbir, 2016b). Apart from valence, words were matched in terms of other factors known to influence EST performance, namely concreteness, frequency of appearance in the language, and length of each word (Burt, 2002; Larsen et al., 2006).

### Behavioral Results

In general, the behavioral results followed the pattern found in earlier behavioral EST research (Imbir, 2016b): response latencies were longer for highly arousing words than for moderately arousing words (Compton et al., 2003; Dresler et al., 2009). This effect can be explained in a way that is consistent with standard EST phenomena (Watts et al., 1986; McKenna and Sharma, 1995, 2004; Thomas et al., 2007). In simple terms, more arousing words are more effective at capturing attention and thus the automated process of reading and accessing the meaning of such words interferes more with the explicit task of naming the color in which they are displayed. Lack of difference in reaction latencies for low arousing words may be accounted for by the stimuli selection. In order to create orthogonally crossed factorial manipulation we had to choose relatively moderate arousing stimuli (cf. word properties section), thus, in our manipulation, low arousing words do not differ from moderate arousing words in causing reaction latencies slowdown (**Figure 4C**). We also found a main effect of subjective significance: response latencies were shorter for highly or minimally subjectively significant stimuli than for moderately subjectively significant stimuli (**Figure 4F**). One explanation of this effect is depicted in **Figure 1**. According to this account, the Stroop task involves two types of processing, the automated processes of reading and accessing the meaning of words and the controlled processing which underpins the naming of the color in which the words are printed. The automated process disrupts the controlled process. Taking into account the duality of the activation mechanisms, more arousing words would be expected to enhance automated processing, while controlled processing would be influenced by the subjective significance of the words (Imbir, 2016b).

In earlier behavioral studies (Imbir, 2016b), no main effects (neither arousal nor subjective significance) were found. But, the patterns of differences for the main effect of subjective significance revealed in the current experiment was also found in the case of the simple main effect for highly arousing stimuli, in the group most susceptible to the modulating role of reflective activation. The presence of both main effects is probably due to the fact, that in comparison to behavioral studies (Imbir, 2016b), current EEG procedure involved three repetitions of a 135-word list presentation, thus reaction latencies were calculated on the basis of a higher number of trials (it was 3 × 15 for a single experimental group, instead of 15 in Imbir, 2016b). Therefore, errors of measurement were lower. It is worth highlighting that the direction of differences in main effects is consistent in both behavioral (Imbir, 2016b) and EEG studies.

Based on behavioral studies (Imbir, 2016b), we predicted that the effect of arousal would interfere with the subjective significance effect. For moderate levels of subjective significance, we may expect a pure replication of arousal effects; namely increasingly arousing stimuli would result in an increasing slowdown in reaction latencies. The presence of subjective significance should modify this relationship in a way that the slowdown of reaction latencies would have to be reduced (due to the activation of resources needed for the controlled-processing dependent explicit Stroop task). Based on behavioral results (Imbir, 2016b), the reduction should occur in both high and low subjective significance levels conditions. For stimuli of low subjective significance, we expected that the slowdown would be caused incidentally. Namely low subjective significant stimuli are perceived as NON-significant, but negation is a form of reflective operation (Deutsch et al., 2006), that needs time to be executed and understood. The long-enough time is not provided by the nature of the Stroop task paradigm requiring fast answers. If so, effects of detected negation should be observed later in time, after the stages of processing that are crucial for cognitive control.

In both studies, we identified the interaction of arousal and subjective significance. Basically, the patterns of results are similar and comparable for each of nine-word group conditions. The longest reaction times were observed for highly arousing words of medium subjective significance level in comparison to most of other conditions (cf. **Table 3**). This allows us to interpret the interaction obtained as a reduction of reaction times to highly arousing stimuli by the presence of high subjective significance. Such simple contrast difference was found in Experiment 2, but not in Experiment 1, in Imbir’s (2016b) early behavioral experiments.

### Electrophysiological Results: Arousal

In this study, the effects related to the arousal level differences of the stimulus words were not localized to any particular brain region. The P2 component (150–290 ms) was sensitive to the arousal level differences of the stimulus words. As **Figure 4B** shows, the amplitude of this component was greater for highly arousing stimuli than for moderately arousing stimuli. This pattern of P2 amplitudes corresponds exactly to the pattern of response latencies (cf. **Figure 4C**), suggesting that processing during the 150–290 ms time window is crucial to the impact of arousal on interference control. P2 is considered to be related to the sensory properties of stimuli (Thomas et al., 2007) and is associated with automatic capturing of attention by stimuli (Van Hooff et al., 2008). However, it has also been found to be sensitive to valence in word processing tasks (Schapkin et al., 2000; Carretié et al., 2004; Herbert et al., 2006; Huang and Luo, 2006).

### Electrophysiological Results: Subjective Significance

There were three ERP components, which were sensitive to the subjective significance of stimulus words. The amplitude of the first ERP component, occurring 50–150 ms after stimulus onset was greater (i.e., more negative) for highly significant stimuli than for minimally or medium significant stimuli. The early emotional ERP effects around 100 ms in word recognition are discussed to index initial attentional resource allocation to process quickly potentially meaningful information (Citron, 2012; Briesemeister et al., 2014). The first explanation we can offer for this rather surprising effect is that the blocked presentation of stimuli created an expectation about the type of stimuli which would be presented which was driven by controlled processing. We suggest that activation resulting from presentation of highly significant stimuli lasted longer than in other cases and thus facilitated the processing of another highly significant stimulus. Some experiments suggest that early effects may be related to relatively complex factors, like word connotations with the discrete basic emotion of happiness (Briesemeister et al., 2014), but not with general positive affect seen from a dimensional point of view. Briesemeister et al. (2014) argue, that such results indicate a conditioned response to categorical rather than dimensional affective information. It is possible that the highly subjective significant stimuli chosen were in fact categorically treated by the participants (in the same way as the NON-significant stimuli).

Alternatively, such an early effect may be explained by some unexpected differences in word properties, like the shapes of the letters in the words or differences in familiarity with the words. Early effects of emotional words were found earlier, but mostly to the well-known stimuli (e.g., the word STOP) processed as a whole, rather than as a standard word stimulus (Hofmann et al., 2009). It is important to highlight, that in the study by Hofmann et al. (2009) the very early responses to words were found to be sensitive to arousal differences. Highly arousing positive as well as highly arousing negative words were found to elicit greater negative potential in the 80–120 ms after the stimulus onset in the paradigm of Lexical Decisions.

The P2 component (150–290 ms) was also sensitive to the subjective significance of the stimuli and once again the pattern of amplitude changes corresponded almost exactly to the pattern of response latencies. Larger P2 responses were associated with slower behavioral responses, suggesting that highly subjectively significant words evoked a different cortical response from moderately subjectively significant stimuli. Thomas et al. (2007) suggested that P2 amplitude might be a more sensitive measure of inhibitory control than behavioral responses. Our results support this claim. Taking into account that the threatening words used by Thomas et al. (2007) differed with respect to their arousal as well as their valence (threat elicits both negative affect and a state of high arousal) it is reasonable to suggest that both the valence and activational properties of stimuli modulate the amplitude of the P2 component.

The last component to show sensitivity to subjective significance was the N450 component (375–530 ms). Minimally significant stimuli elicited larger N450 potential than moderately or highly significant stimuli. We may treat this pattern of results as the manifestation of the effects of pure significance factor, postulated in **Figure 1**. In previous studies, the N450 component has been associated with higher order processes such as conflict detection (West, 2003) or selection among competing responses (West and Alain, 1999). In the Stroop task, the N450 potential may reflect conflict resolution processes and the inhibition of the semantic representation of stimulus words in the incongruent condition (West and Alain, 1999, 2000). In EST paradigms, the N450 was found to differentiate according to behavioral responses (Van Hooff et al., 2008). These authors suggested that the N450 component varies according to the activation of the neural system responsible for suppressing conceptual representations. Our results suggest that the N450 potential reflects the conceptual processing involved in negation processing. It is probable that the differences in amplitude reflect the detection of a low level of subjective significance associated with certain stimuli, thus leading to the urge to avoid such objects in order not to disturb one’s goals.

It is worth highlighting that no interaction between arousal and subjective significance was found in the case of amplitude of evoked potential in all time windows analyzed, especially in the P2 component. The lack of this interaction is understandable when only one factor modulates amplitudes (cf. early and late effects), but such a pattern should be present in a component found to resemble behavioral differences. In **Table 5** we summarized the simple contrast for each of the nine conditions of the study. As one can see, the pattern of difference resembles closely the pattern shown in **Table 3**. Therefore, although there was no interaction effect found for arousal and subjective significance in the P2 component, the behavioral and amplitude results correspond with each other quite strictly. This supports our claim that P2 component activity of the cortex is crucial for behavioral phenomena in modified emotional Stroop task.

**Table 5:** Average amplitude of P2 component (in μV) for each condition.

		Arousal			
		Low *M* (*SEM*)	Medium *M* (*SEM*)	High *M* (*SEM*)	
Significance	Low	3.20 (0.28)	2.53 (0.28)	3.16 (0.31)	2.96 (0.17)
	Medium	3.11 (0.32)	3.00 (0.32)	3.71 (0.29)	3.27 (0.18)
	High	2.65 (0.25)	2.77 (0.33)	2.98 (0.32)	2.80 (0.17)
	Total	2.98 (0.16)	2.77 (0.18)	3.28 (0.18)	3.01 (0.10)

*The shading illustrates the greatest differences between conditions measured by simple contrasts. The amplitude in condition marked in dark gray was higher than the amplitudes in conditions marked in light gray (please compare with **Table 3**).*

## Conclusion

The results of this study corroborate the proposed model of dual activation mechanisms. Once more (cf. Imbir, 2016b), the behavioral data indicate that the arousal level of stimuli influences cognitive control in the modified Stroop task. The electrophysiological data shed new light on the interpretation of current and earlier behavioral results. The pattern of ERP amplitude differences found during the execution of the modified Stroop task suggests that the P2 component is critical to the behavioral response, as the P2 electrophysiological response and behavioral reaction time data followed exactly the same pattern. We also found that early ERP components were sensitive to highly significant stimuli and later ERP components were sensitive to minimally significant stimuli. In other words, subjective significance appears to influence the electrophysiological response at various stages of processing. The rational and controlled mind activation could, in fact, work in a time specific way, manifested here as the attentional effect of preparedness or facilitation of processing for the next subjectively significant stimulus (N1) and could be directly manifested in later stages of processing (N450).

## Author Contributions

All authors contributed to final version of the manuscript. Theoretical proposition: KI; Design: KI and JŻ; Method (words): KI; Method (EEG measures) JŻ and TS; Experimental procedure programming: TS and JŻ; Experiment execution: TS and JŻ; Statistical analyses: JŻ, KI, TS, JD, and GB; Results description: JŻ and JD; Results discussion: KI; Figures: JŻ, TS, KI, JD, and GB.

## Conflict of Interest Statement

The authors declare that the research was conducted in the absence of any commercial or financial relationships that could be construed as a potential conflict of interest.

## Footnotes

1 The trigger consisted of a circuit measuring the brightness of a small rectangular portion of the screen, which was covered by the trigger. The brightness of that part of the screen was changed at stimulus onset. The signal from the circuit was recorded, together with the EEG signal, on an auxiliary input of the amplifier. This signal was later used to extract and align trial data.

2 Biomedical Signal Viewer, Analyzer and Recorder on GPL (SVAROG) developed at the Department of Biomedical Physics, University of Warsaw (http://svarog.pl/).
