# Supplementary material for: Two Aspects of Activation: Arousal and Subjective Significance – Behavioral and Event-Related Potential Correlates Investigated by Means of a Modified Emotional Stroop Task
Source: Front Hum Neurosci. 2017 Dec 12;11:608. doi: 10.3389/fnhum.2017.00608 (PMC5732992; doi:10.3389/fnhum.2017.00608)
Supplement: Supplementary file 2 [file Data_Sheet_2.docx]

**Appendix 2. Additional ERP analysis**

In order to give a broader perspective on the electrophysiological reactions to the stimuli we computed grand average ERP and vEOG across subjects and repetitions of given level of arousal (Fig. A2.1), and given levels of subjective significance (Fig. A2.2). In this way, a topographical distribution of components can be observed.

| 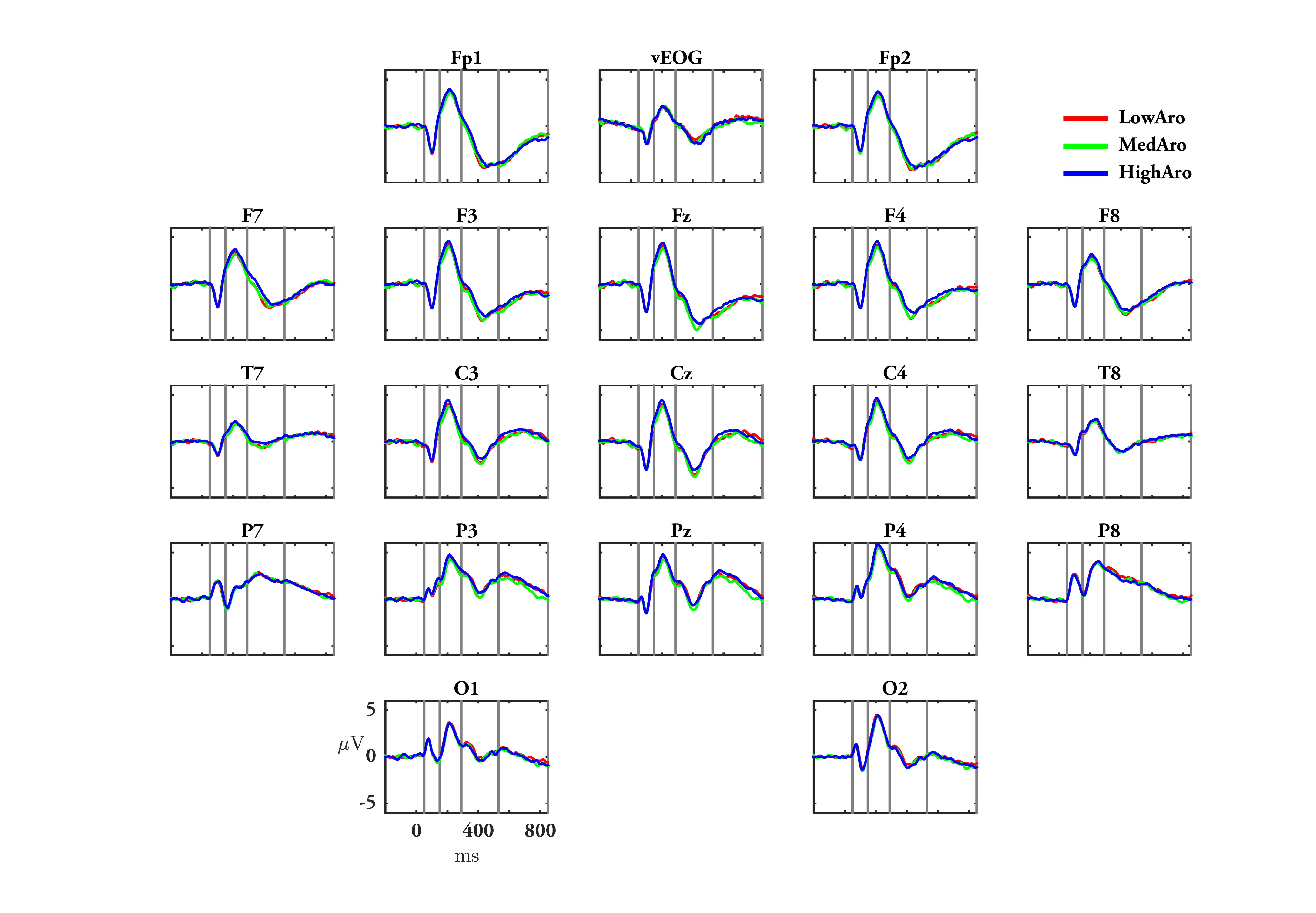 |
| --- |
| **Figure A2.1.** Time courses of grand average ERP on each of the EEG electrodes, and vertical EOG for the three levels of arousal factor. Conditions are marked in colours: red – low arousal, green – medium arousal, blue - high arousal. Horizontal axis time in ms, vertical axis amplitude in μV. Vertical grey lines mark the borders of the time windows used in the analysis. |

| 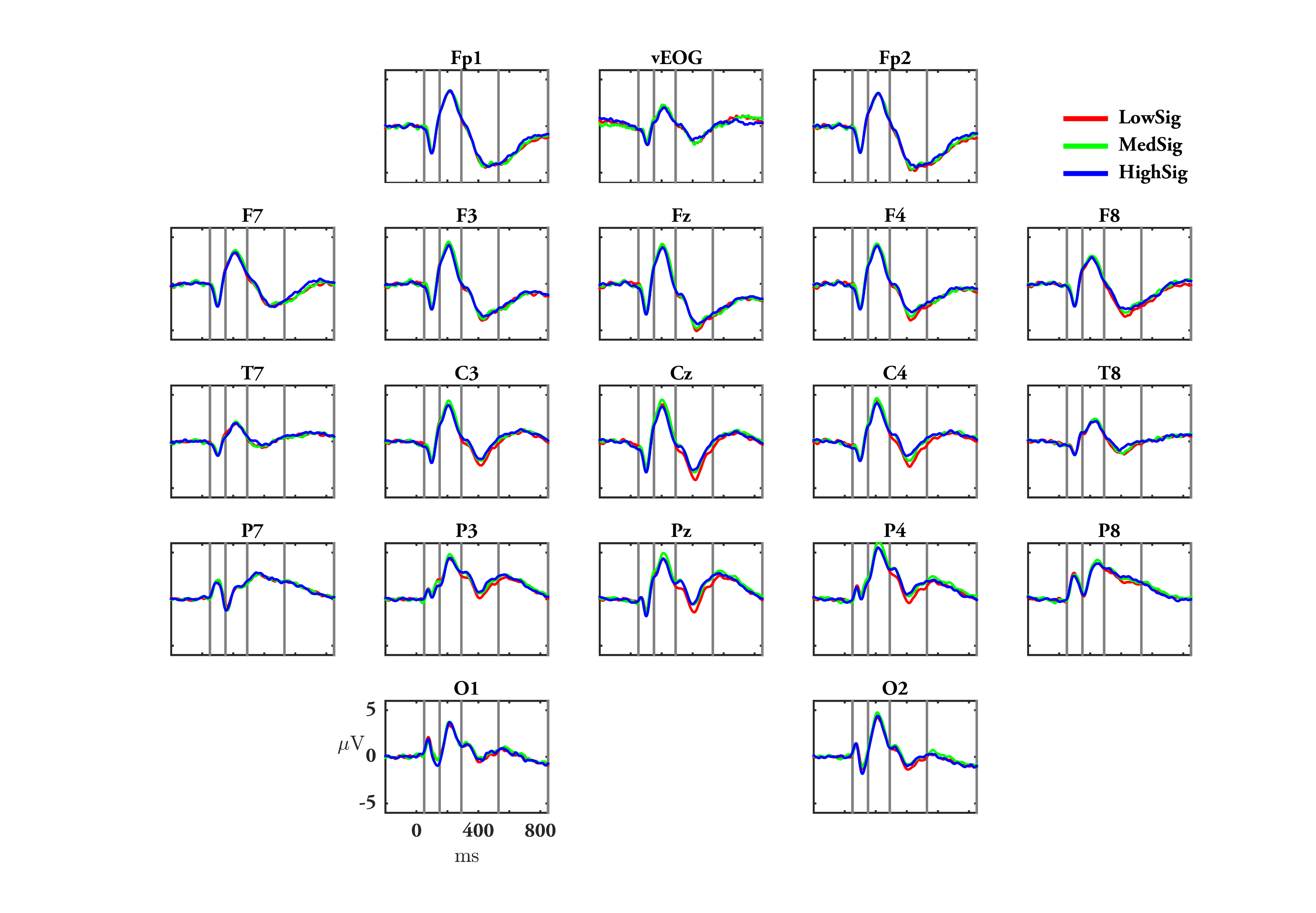 |
| --- |
| **Figure A2.2.** Time courses of grand average ERP on each of the EEG electrodes, and vertical EOG for the three levels of subjective significance factor. Conditions are marked in colours: red – low significance, green – medium significance, blue - high significance. Horizontal axis time in ms, vertical axis amplitude in μV. Vertical grey lines mark the borders of the time windows used in the analysis. |

In order to further visualise the distribution of differences between the levels of the factors under consideration, we computed the topographical maps of contrasts (Fig. A2.3 and A2.4).

| -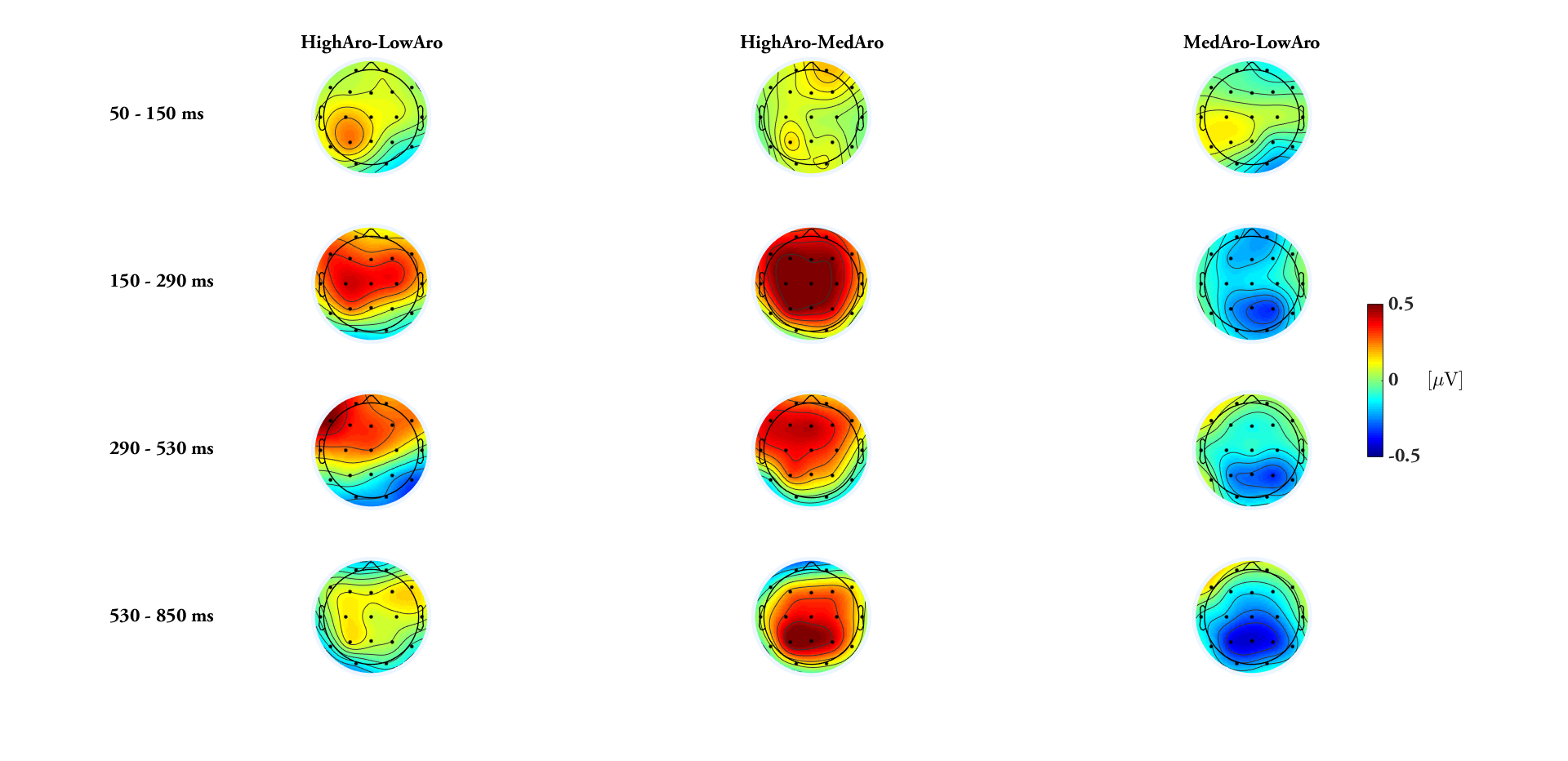 |
| --- |
| **Figure A2.3.** Topographical distributions of the ground average ERP contrasts between levels of arousal factor averaged within analysed time windows. Each subplot displays the distribution of the contrast named at the top of each column and for the time window indicated at the left of the row. Amplitude difference is color-coded according to the scale on the right. |

| 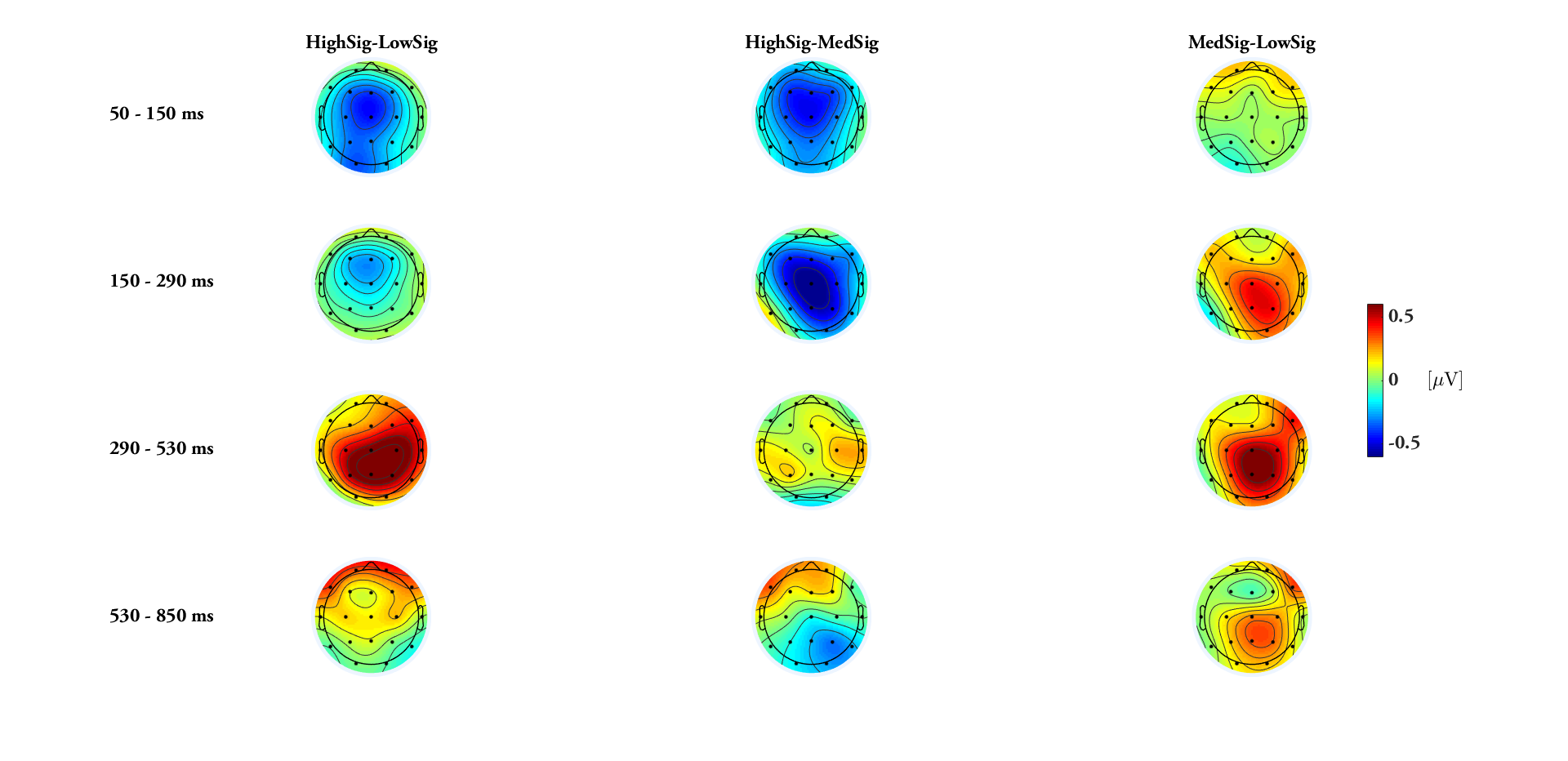 |
| --- |
| **Figure A2.4.** Topographical distributions of the ground average ERP contrasts between levels of significance factor averaged within analysed time windows. Each subplot displays the distribution of the contrast named at the top of each column and for the time window indicated at the left of the row. Amplitude difference is color-coded according to the scale on the right. |
